# The β-NAD^+^ salvage pathway and PKC-mediated signaling influence localized PARP-1 activity and CTCF Poly(ADP)ribosylation

**DOI:** 10.18632/oncotarget.19841

**Published:** 2017-08-03

**Authors:** David J.P. Henderson, JJ L. Miranda, Beverly M. Emerson

**Affiliations:** ^1^ The Salk Institute for Biological Studies, La Jolla, CA, USA; ^2^ Department of Cellular and Molecular Pharmacology, University of California, San Francisco, CA, USA

**Keywords:** Poly(ADP)ribosylation, CTCF, NMNAT-1, β-NAD+, PARP-1

## Abstract

Poly(ADP)ribosylation (PARylation) of the chromatin architectural protein CTCF is critical for CTCF-dependent regulation of chromatin boundary and insulator elements. Loss of CTCF PARylation results in epigenetic silencing of certain tumor suppressor genes through destabilization of nearby chromatin boundaries. We investigated the metabolic and mechanistic processes that regulate PARP-1-mediated CTCF PARylation in human cancer cell lines and discovered a key role for the expression and activity of β-NAD+ salvage enzymes, NAMPT and NMNAT-1. These enzymes are downregulated in cells that exhibit reduced CTCF PARylation, resulting in a decreased concentration of nuclear β-NAD+. In these cells, decreased NMNAT-1 expression is enforced by a proteasome-mediated feedback loop resulting in degradation of NMNAT-1, transcriptional repression of NAMPT, and suppression of PARP-1 activity. Interestingly, dePARylated CTCF is associated in a stable protein complex with PARP-1 and NMNAT-1 in cancer cells harboring silenced tumor suppressor genes. Although the metabolic context in these cells favors suppression of PARP-1 activity, CTCF PARylation can be restored by Protein Kinase C (PKC) signaling. PKC induces dissociation of the catalytically inactive PARP-1/NMNAT-1/CTCF protein complex and phosphorylation of NMNAT-1, which stimulates its proteasome-mediated degradation. Our findings suggest that CTCF PARylation is underpinned by a cellular metabolic context engendered by regulation of the β-NAD+ salvage pathway in which NMNAT-1 acts as a rheostat to control localized β-NAD+ synthesis at CTCF/PARP-1 complexes.

## INTRODUCTION

Poly(ADP)ribosylation (PARylation) of transcription factors and chromatin components regulates critical cellular processes such as DNA repair, mitosis and many features of chromatin structure and architecture [1-5]. Indeed, PARylation of chromatin regulators changes their cofactor interaction specificity and directly affects nucleosomal structure by modifying core histone components [3, 6-8]. Of particular interest is PARylation of the multi-functional DNA binding protein CTCF, which regulates its activity at chromatin boundaries and genetic insulators as well as epigenetic imprinting [9, 10].

CTCF is a functionally diverse nuclear protein that regulates critical aspects of chromatin structure [5, 11, 12]. CTCF binding at epigenetic boundaries and insulator elements underpin the organization of domain-specific hetero- and eu-chromatin [12, 13] and long-range promoter-enhancer interactions [14-16]. CTCF also acts as a scaffolding protein conferring 3D chromatin organization [11, 12]. Due to its role in chromatin structure and function, CTCF is a critical transcription factor for many genes where it interacts with core promoters, key enhancer elements, regional boundary and insulator sites [9, 12, 17]. In view of its multifaceted relationship with nuclear function, it is important to understand the mechanistic basis of CTCF post-translational modifications and their impact on DNA binding and cofactor association, as these features specify its activities at different genomic locales.

Modification of CTCF by poly(ADP)ribosylation (PARylation) has been shown to confer selective binding of cofactors to form specific CTCF protein complexes that establish and maintain critical features of chromatin structure [9, 18, 19]. Previous work in human breast cancer cell lines showed that decreased CTCF PARylation is accompanied by pronounced changes in cofactor interactions within CTCF complexes and destabilization of specific CTCF-dependent chromatin boundaries, resulting in epigenetic silencing [9, 19]. Intriguingly, a mutually exclusive binding relationship between CTCF, Nucleolin and PARP-1 within multiprotein CTCF complexes is dependent upon the PARylation status of CTCF. In cells with reduced CTCF PARylation, the stable association of PARP-1 with CTCF protein complexes occurs as a reaction intermediate, thereby highlighting a block in the productive modification of CTCF [19]. This enzymatic inhibition suggests that an undefined mechanism exists that modulates CTCF PARylation, which may significantly impact epigenetic organization and chromatin architecture through PARylation-dependent CTCF functions.

To decipher the mechanisms that regulate CTCF PARylation, we examined the enzymology underpinning PARP-1 activity. Specifically, how enzymes of the β-NAD^+^ salvage pathway interact with poised PARP-1/CTCF protein complexes in cells with dePARylated CTCF. Nicotinamide Phosphoribosyltransferase (NAMPT) and Nicotinamide Mononucleotide Adenylyltransferase 1 (NMNAT-1) are essential enzymes that catalyze the final two reactions of the nuclear β-NAD^+^ salvage pathway and are of underlying importance to nuclear PARylation. Here we show that a purposeful suppression of PARP activity correlates with down-regulation of these enzymes at the transcriptional and post-translational levels. Furthermore, we show how enzymatically inactive PARP-1/CTCF complexes bind NMNAT-1 in a poised state, and that disruption of this complex through Protein Kinase C activation leads to the phosphorylation and degradation of NMNAT-1 and concurrent CTCF PARylation.

To further investigate the role of NMNAT-1 at targeted protein complexes we reconstituted the enzymatic reaction with CTCF *in vitro* and demonstrate how NMNAT-1 stimulates PARylation by synthesizing β-NAD^+^. We also show that in certain metabolite contexts NMNAT-1 facilitates localized pyrophospholytic cleavage of β-NAD^+^, resulting in *in vitro* suppression of CTCF PARylation. Taken together, these cell-based and biochemical assays reveal that the β-NAD^+^ salvage pathway can underpin protein-specific PARylation and that CTCF PARylation is regulated by a complex network of regulatory events. Our findings highlight a novel enzymatic mechanism that may modulate distinct CTCF nuclear activities.

## RESULTS

### Reduced PARylation activity in cells with defective CTCF PARylation is correlated with down-regulation of enzymes in the β-NAD^+^ salvage pathway, but not β-NAD^+^ availability

**Figure 1:**
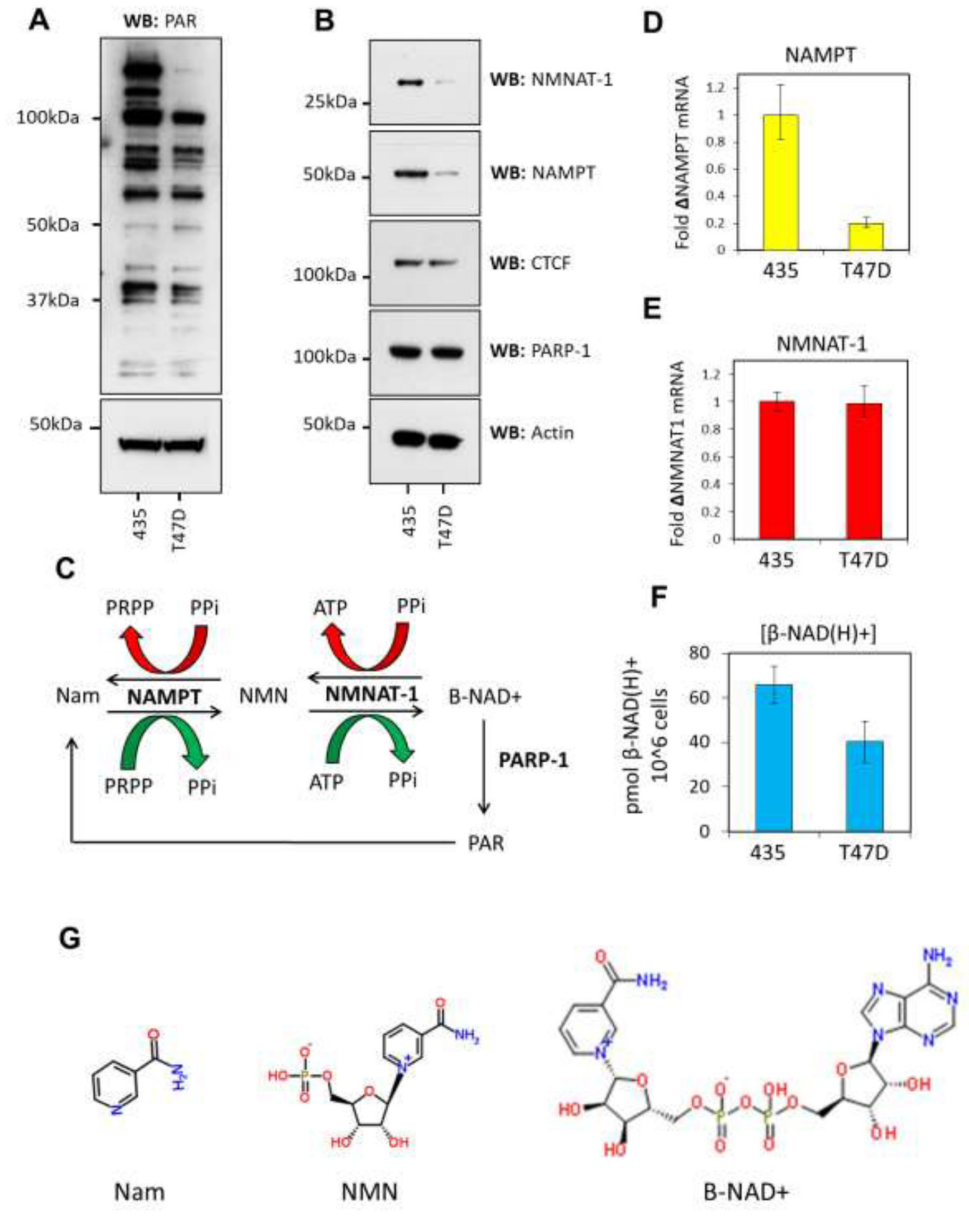
The β-NAD^+^ salvage pathway is down-regulated in T47D cells **A.** Western blots showing PARylated proteins in 435 vs T47D cells. 25μg of nuclear lysates were analyzed and actin used as a loading control. 435 cells exhibit significantly more PARylation than T47D cells as shown by the decrease in number and intensity of protein bands reacting with a PAR-specific antibody. **B.** Expression of NMNAT-1 and NAMPT was assessed by western blot. Both proteins are down-regulated in T47D cells when compared to 435 cells. CTCF and actin are used as equal loading controls. **C.** A schematic of NAMPT and NMNAT-1 activity in the β-NAD^+^ salvage pathway. The forward (β-NAD^+^ synthesis) reaction products are shown by a green arrow and the reverse reaction indicated by a red arrow. **D.** RT-qPCR showing NAMPT mRNA levels are down-regulated in T47D cells. Results are shown as a fold-change compared to levels in 435 cells. **E.** RT-qPCR data show equivalent expression of NMNAT-1 mRNA in T47D cells. Results are shown as a fold-change compared to 435 cell levels. **F.** β-NAD(H)^+^ levels are reduced globally in T47D cells compared to 435 cells as assessed by alcohol dehydrogenase-coupled MTT assay for β-NAD(H)^+^. **G.** The chemical structures of nicotinamide (Nam), Nicotinamide mononucleotide (NMN) and β-NAD^+^.

It has been shown previously that CTCF PARylation is reduced in T47D human breast cancer cells that contain an epigenetically silenced *p16*^*INK4A*^ tumor suppressor gene and loss of an upstream CTCF-regulated chromatin boundary when compared to MDA-MB-435 (435) breast cancer cells [19]. To gain mechanistic insight into the regulation of CTCF PARylation in this system we first compared the nuclear profile of PARylated proteins between T47D and 435 cells (Figure 1A). In so doing we hoped to define the global context of PARP activity and contrast this with CTCF dePARylation. Sub-cellular fractionation followed by western blotting for PAR moieties in nuclear lysates revealed that the levels of protein PARylation are significantly reduced in T47D cells when compared with 435 cells. We observed no change in PARP-1 protein abundance between the two cell lines (Figure 1B) and therefore concluded that modulation of PARP activity underlies the observed deficit in protein PARylation in T47D cells. Of note, these cell lines do not exhibit the 180kDa form of CTCF which has previously been linked to PARylation (as assessed by western blot for CTCF using monoclonal (Figure 1B) and polyclonal (Supplementary Figure S1) CTCF antisera). This observation may reflect the mode of CTCF PARylation in advanced breast cancer [20]. However, PARylation of the 130kDa form and the molecular regulation thereof remains pertinent to its boundary function and epigenetic regulator status.

β-NAD^+^ is the prerequisite metabolite required for PARylation and the regulation of its synthesis at protein complexes underpins PARP activity [21]. The β-NAD^+^ salvage pathway synthesizes the majority of β-NAD^+^ within the nucleus [22]. Of the enzymes which catalyze the reactions in this pathway, NAMPT and NMNAT-1 represent the rate-limiting and final enzymatic steps in the synthesis of β-NAD^+^ [21, 23] (Figure 1C, 1G). Indeed, NMNAT-1 has previously been shown to stimulate PARylation through its binding to PARP-1 [24]. NAMPT and NMNAT-1 protein and mRNA expression levels were assessed in 435 and T47D cells to ascertain if differential regulation of expression might contribute to the observed reduction in PARP activity. Western blot analysis showed that the protein expression of both these key enzymes is significantly reduced in T47D cells (Figure 1B). This reduction in protein expression was mirrored at the mRNA level for NAMPT (Figure 1D), indicating that a transcriptional or mRNA processing-mediated mechanism leads to decreased protein abundance. NMNAT-1 mRNA levels remained stable between the two cell lines (Figure 1E), indicating that the enzyme is differentially regulated during translation or at the post-translational level. In support of these observations we found that the levels of β-NAD(H)^+^ in T47D cells were significantly reduced compared to that observed in 435 cells (Figure 1F). Down-regulation of NAMPT and NMNAT-1 coupled with decreased β-NAD(H)^+^ levels suggest that availability of the enzymes at active enzymatic complexes within the nucleus could be a passive limiting factor affecting protein PARylation in T47D cells by decreasing PARP-1 stimulation and reducing β-NAD^+^ supply.

**Figure 2:**
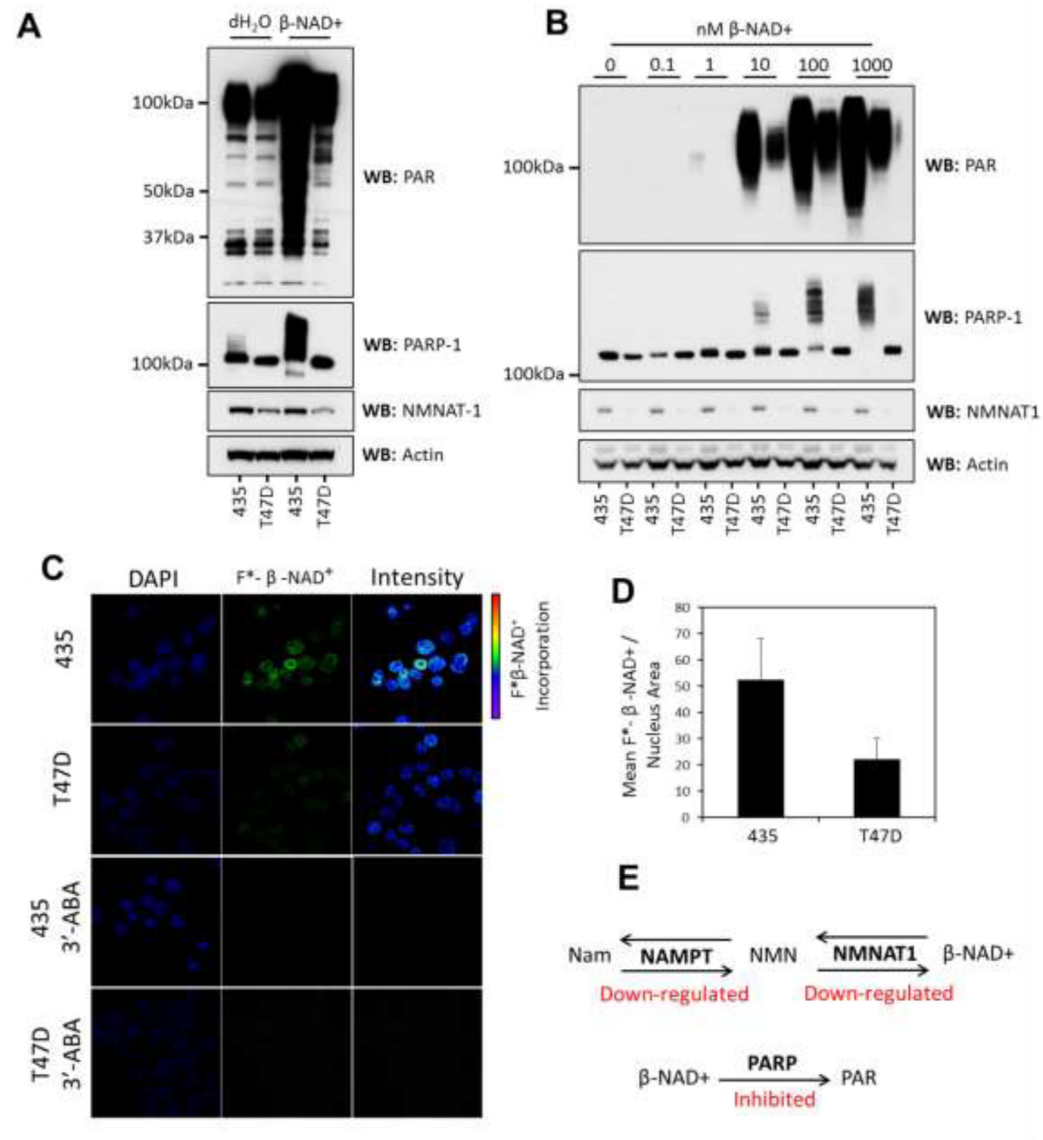
PARylation is inhibited in T47D cells **A.** *In vitro* PARP response assay showing that addition of β-NAD^+^ (1mM) to 100µg of nuclear lysates (1µg/µl) results in greater synthesis of PAR in lysates from 435 cells when compared to T47D cells. PARP-1 auto-modification and increasing molecular weight correlates with elevated PARylation; actin was used as an equal loading control. **B.** A dose response showing that increasing concentrations of β-NAD^+^ result in increased amounts of PARylation in both 435 and T47D cells but the relative suppression of PAR synthesis in T47D cells is maintained; actin was used as a loading control. **C.** Fluorescently labeled β-NAD^+^ incorporation into isolated cell nuclei shows increased incorporation in 435 cells when compared to T47D. Inhibition of PARP activity using 3’-ABA resulted in the ablation of fluorescent metabolite incorporation confirming that incorporation was due to PARP activity. The reproducibility of this observation between cell lysates and cell nuclei indicates that the spatial context of the nucleus and the presence of genomic chromatin did not significantly alter the observed change in PARP activity. **D.** Quantification of fluorescence incorporation in 435 and T47D cell nuclei (55:22 mean relative fluorescence units/nucleus area measured 435:T47D (p<0.05)). **E.** Graphic depicting the coordinated inhibition of PARylation and β-NAD^+^ synthesis in T47D nuclei. Nam = nicotinamide, NMN = nicotinamide mononucleotide.

To investigate if β-NAD^+^ availability limits PARylation in T47D nuclei we performed *in vitro* PARP response reactions where exogenous β-NAD^+^ was supplied to nuclear lysates. The resulting PAR synthesis was visualized and assessed by western blotting (Figure 2A). A significant amount of PAR was synthesized in 435 nuclear lysates in contrast to T47D samples where only limited PARP activity was observed. PARP-1 auto-modification also followed this pattern as measured by the increase in PARP-1 molecular weight. These results show that inhibition of PARP activity in T47D cells is maintained in a post-lysis reaction environment where β-NAD^+^ is in excess. We also investigated if varying concentrations of β-NAD^+^ (0 mM-1 mM) would interact differently with PARP complexes in the nuclear lysates (Figure 2B). We found that the differential PARP activity observed between 435 and T47D cells is maintained at increasing concentrations of β-NAD^+^, but also noted that increasing PARP activity was stimulated by β-NAD^+^ in a dose-dependent manner in both cell lines, indicating a potential competitive inhibition or suppression of activity in T47D cells. We surmise that PARP activity is suppressed by the presence or absence of a cofactor or post-translational modification rather than being solely limited by metabolite abundance due to down-regulation of the β-NAD^+^ salvage pathway.

To confirm that the difference in PARP activity was not due to the changing context of protein interaction or DNA binding during cell lysis, we examined PARylation in isolated cell nuclei using fluorescently labeled β-NAD^+^. By incubating the isolated nuclei with a mixture of labeled and unlabeled β-NAD^+^ the resulting PAR chains were metabolically labeled by incorporation of the fluorescent moiety. Using confocal microscopy we examined the localization of fluorescently labeled PAR chains *in situ* (Figure 2C). We found that PARP enzymes incorporated on average around 50% less labeled metabolite in T47D cells (Figure 2D). This confirmed that PAR synthesis is actively suppressed in these cells, compounding the reduction in β-NAD^+^ availability imposed by the down-regulation of NAMPT and NMNAT-1 (Figure 2E).

### Reduced PARylation efficiency results in stabilization of an enzymatically non-productive CTCF/PARP-1/NMNAT-1 complex

After establishing that PARP activity is suppressed in T47D nuclei, we then analyzed the basis for interaction between PARP-1 and CTCF, since inactive PARP-1 associates with CTCF in a poised reaction complex in T47D but not 435 cells [19]. Due to the striking down-regulation of NMNAT-1 and the β-NAD^+^ salvage pathway we investigated if NMNAT-1 is bound to active or inactive CTCF complexes and how modulation of its binding might affect CTCF PARylation.

**Figure 3:**
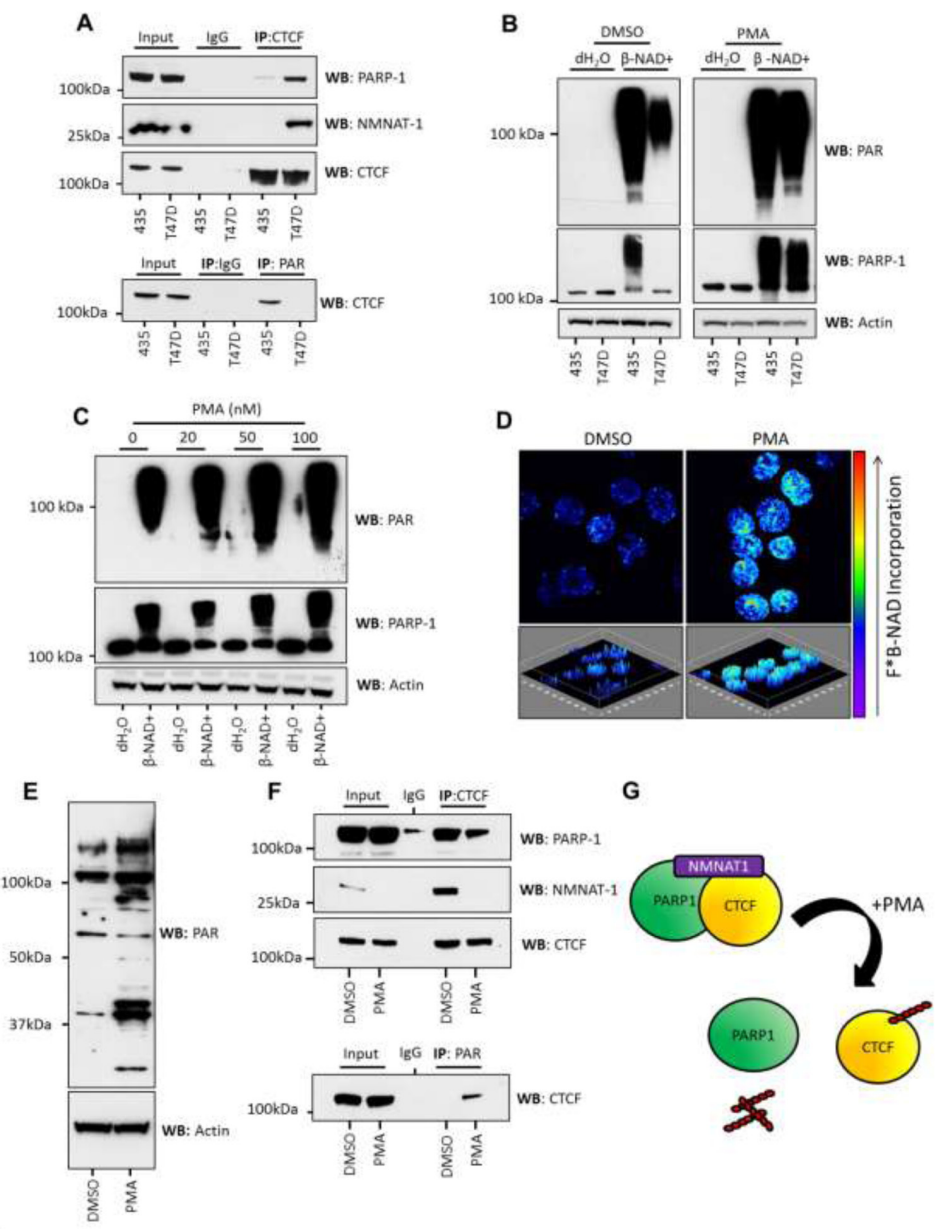
PMA stimulation of PARylation in T47D cells is accompanied by the loss of NMNAT-1 from inactive CTCF/PARP-1 protein complexes **A.** NMNAT-1 binds to CTCF/PARP-1 complexes in T47D cells but not in 435 cells, as assessed by IP of CTCF and western blotting for PARP-1 and NMNAT-1 (upper panel). This association inversely correlates with PARylation of CTCF (lower panel) assessed by PAR IP and western blot for CTCF. **B.** 435 and T47D cells were treated with 100nM PMA and β-NAD^+^ (1mM) was added to 100µg of nuclear extract (1μg/μl). PMA treatment increased PARP activity in T47D cells to the same level as observed in 435 cells as assessed by PAR synthesis (upper panel) and PARP-1 auto-modification as determined by increasing molecular weight (lower panel). Actin was used as a loading control. Direct addition of PMA to nuclear lysates had no effect on PARP response (Supplementary Figure S3). **C.** The observed increase in PARP response in T47D cells was dose-dependent upon the concentration of PMA added to the cells. Actin was used as a loading control. **D.** The increase in PAR synthesis observed in T47D cells treated with PMA (100nM) was assessed in isolated cell nuclei. Elevated PARP activity was reproduced in cell nuclei as viewed by the increase in incorporation of fluorescently labeled β-NAD^+^. Upper panels represent a single image of a z-stacked composite image, which is shown in the lower panels and can be viewed as media files (*.avi - Supplementary Media File 1 and Supplementary Media File 2). Fluorescent PAR imaging of DMSO and PMA-treated T47D nuclei can be seen in Figure 3 and Supplementary Figure S2. **E.** PMA treatment resulted in an increase in PARylated proteins observed in T47D nuclear lysates. **F.** PMA treatment also led to the reduction of PARP-1 and NMNAT-1 binding to CTCF protein complexes in T47D cells (upper panel) and a concurrent increase in CTCF PARylation (lower panel). Interestingly, NMNAT-1 was observed to decrease in abundance upon PMA stimulation. This was confirmed in several human breast cancer cell lines (data not shown). **G.** Graphic depicting the formation of stable CTCF/PARP-1/NMNAT-1 protein complexes and subsequent PMA-induced PARylation of CTCF and dissociation of the CTCF/PARP-1/NMNAT-1 protein complex.

In T47D cells, CTCF, PARP-1 and NMNAT-1 all localize to the nucleus (immunofluorescence of transfected cells, Supplementary Figure S2). The association of endogenous NMNAT-1 with CTCF in 435 and T47D cells was assessed by immunoprecipitation of CTCF from nuclear lysates. We found that although NMNAT-1 is significantly down-regulated in T47D cells it binds strongly to the enzymatically inactive CTCF/PARP-1 complex (Figure 3A). This protein:protein interaction was not observed in 435 cells. This result is in agreement with a previous study which showed that NMNAT-1 localizes to PARP-1 [24]. However, in this case the proximity of NMNAT-1 is not sufficient to stimulate PARP-1 activity [24] as evidenced by the lack of CTCF PARylation.

### Phorbol-12-Myristate-13-Acetate (PMA) treatment induces PARP activity, restores CTCF PARylation and dissociates the CTCF/PARP-1/NMNAT-1 protein complex

The PKC activator PMA has been shown to increase PARP-1 activity in different cell types. We tested whether treatment of T47D cells with PMA would result in increased PARP activity and concurrent CTCF modification.

We found that treatment of T47D cells with PMA resulted in a significant increase in PARP-1 activity (Figure 3B). PMA-induced increase in PAR synthesis was dose-dependent (Figure 3C) but not observed when the PKC activator was added directly to nuclear lysates (Supplementary Figure S3). Therefore, activation of cytosolic PKC is required to increase PARP-1 activity rather than PMA directly modulating the activity of nuclear factors. We also observed a PMA-induced increase in the *in situ* incorporation of fluorescently labeled β-NAD^+^ within T47D nuclei (Intensity images in Figure 3D, fluorescence images in Supplementary Figure S4).

During fluorescent labeling of nuclear PAR we observed a particular accumulation of fluorescence at the nucleoli of PMA-treated cells together with enhanced PARylation of nucleolin, a major constitutive protein of the nucleolus (Figure 3D, Supplementary Figure S5). 3D imaging of DMSO-treated (Supplementary Media File 1) and PMA-treated cell nuclei (Supplementary Media File 2) show a clear change in amount and distribution of fluorescently labeled PAR. Significantly, insulator elements are known to be tethered to the nucleolus in a CTCF-dependent manner [15] and association of nucleolin with CTCF is dependent on PARylation of each protein, which facilitates the function of CTCF complexes at chromatin boundaries [19]. The increase in general PARylation was confirmed by western blot which clearly showed an elevation of protein PARylation in response to PMA treatment (Figure 3E).

Immunoprecipitation of CTCF complexes from PMA-treated and control cell lysates revealed that PMA induced dissociation of both PARP-1 and NMNAT-1 from CTCF and stimulated CTCF PARylation (Figure 3F). Interestingly, NMNAT-1 expression is further down-regulated in PMA-treated T47D cell lysates. This shows that the global changes in PARP activity are reflected at the level of individual protein targets and that CTCF can be PARylated by PARP-1 in response to an altered cellular signaling program which results in the down-regulation of NMNAT-1 (Figure 3G).

### PMA induces phosphorylation and proteasome-mediated degradation of NMNAT-1 in cells deficient in CTCF PARylation

**Figure 4:**
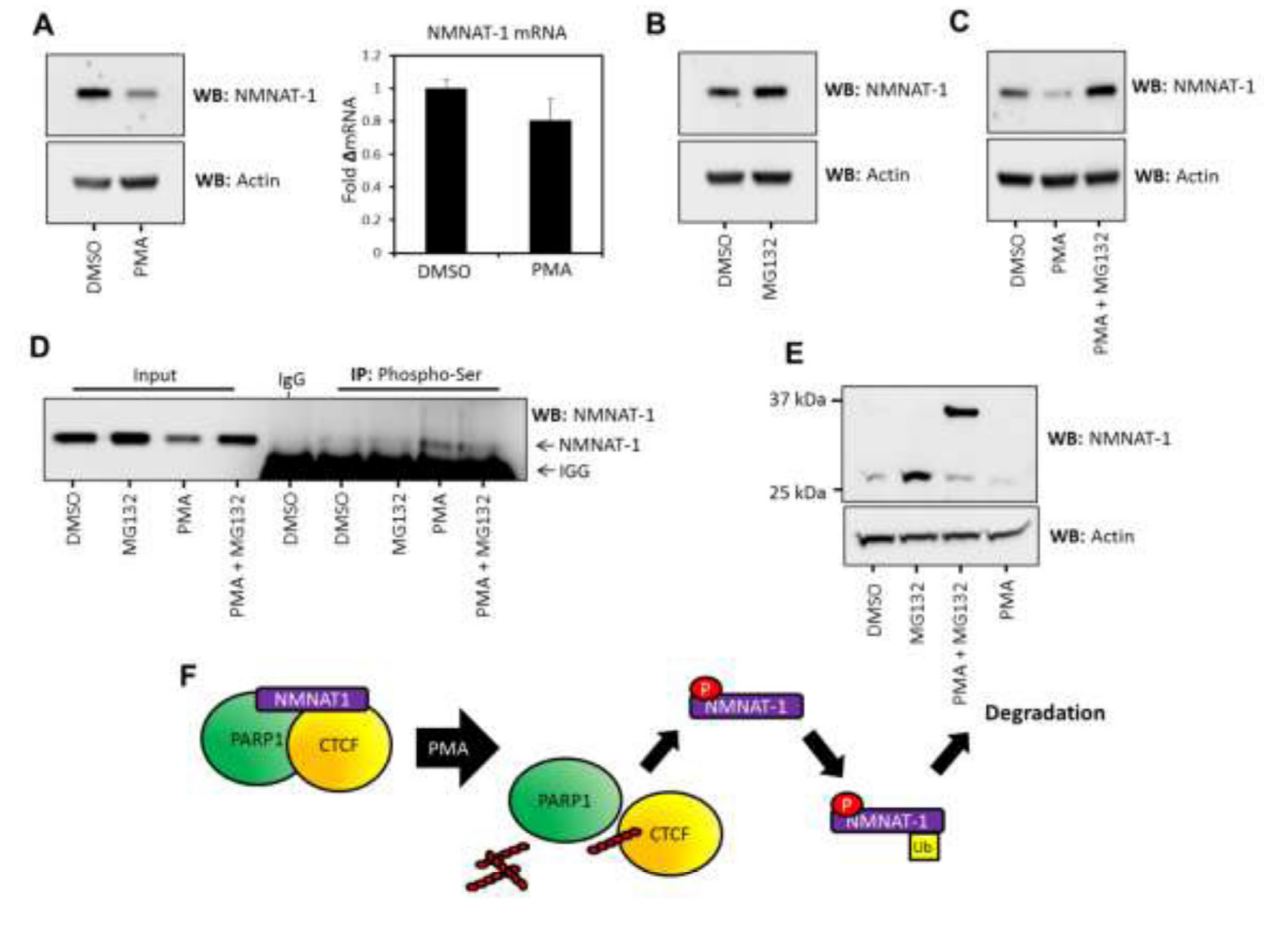
NMNAT-1 expression is regulated at the post-translational level in T47D cells **A.** (*Left*) NMNAT-1 protein expression is down-regulated upon PMA treatment as assessed by western blot. Actin expression was used as a loading control. *(Right)* NMNAT-1 mRNA levels are not significantly altered with PMA treatment. **B.** Inhibition of the proteasome with 1µM MG132 leads to an increase in NMNAT-1 expression in T47D cells. Actin expression is shown as a loading control. **C.** PMA induced down-regulation of NMNAT-1 protein expression is ablated by MG132-mediated proteasome inhibition. Actin expression was used as an equal loading control. **D.** Phospho-serine immunoprecipitation from T47D cells treated with MG132 (1µM), PMA (100nM) or a combination of both treatments. NMNAT-1 phosphorylation can be detected at basal levels in mock and MG132-treated cells, which is increased by PMA treatment. Light chain IgG reactivity is visible at the bottom of the panel due to the reactivity of the antisera used in this experiment. **E.** T47D cells were treated with MG132, PMA or a combination of these agents and analyzed by western blot. Extended (12 hr) combinatorial treatment with PMA (100nM) and MG132 (1µM) led to the accumulation of a weigh-shifted form of NMNAT-1, potentially representing the mono-ubiquitination of the protein (which would lead to an 8kDa shift in MW). This indicates that mono-ubiquitination may be stimulated by PMA-induced phosphorylation of NMNAT-1 and dissociation from bound protein complexes. **F.** Summary graphic illustrating the proposed phosphorylation-regulated, proteasome-dependent degradation of NMNAT-1 in T47D cells.

NMNAT-1 is central to the regulation of the β-NAD^+^ salvage pathway and to protein PARylation. We therefore analyzed the mechanism behind NMNAT-1 down-regulation and how this may affect PARP-1 activity. No significant change in NMNAT-1 mRNA transcript abundance upon PMA treatment was observed (Figure 4A), thereby indicating that down-regulation is likely post-translationally controlled. This is strikingly similar to the down-regulation of NMNAT-1 observed between 435 and T47D cells, and suggests that a mode of targeted degradation controls NMNAT-1 expression in T47D cells.

To test this hypothesis we assessed the phosphorylation status of NMNAT-1 in response to PMA treatment and used the inhibitor MG132 to investigate if the protein is down-regulated by a proteasome-dependent mechanism. We found that inhibition of the proteasome by MG132 treatment increases NMNAT-1 expression in T47D cells (Figure 4B) and inhibits its PMA- induced down-regulation (Figure 4C). As expected, PMA treatment also led to the serine specific phosphorylation of NMNAT-1 (Figure 4D). Extended treatment (12 hr) with a combination of MG132 and PMA induces the appearance of a molecular weight-shifted protein species of NMNAT-1 (Figure 4E). We hypothesize that this is a mono-ubiquitinated protein species due to the molecular weight shift (ubiquitin is 8 kDa) and its stabilization in response to MG132 treatment. The mono-ubiquitination of NMNAT-1 may perform a regulatory function in its nuclear redistribution or activity. Alternatively, mono-ubiquitination may act as a ‘priming’ or intermediate modification state for further poly-ubiquitination and targeting to the proteasome, which is consistent with the sensitivity of NMNAT-1stability to MG132. Importantly, inhibition of the proteasome was observed to increase the abundance of the heavier species of NMNAT-1 only when cells were treated with PMA. This suggests that PKC activation drives an increase in ubiquitination and degradation of NMNAT-1 over and above the basal levels of protein turnover.

When combined with our previous observations these data suggest that NMNAT-1 turnover occurs at a faster rate and is more sensitive to further perturbation in T47D cells compared to 435 cells, accounting for the observed down-regulation. However, a sub-species of NMNAT-1 is held in an inactive conformation with PARP/CTCF protein complexes. The phosphorylation of this NMNAT-1 species leads to its eviction, which allows targeted mono-ubiquitination and, ultimately, proteasomal degradation of NMNAT-1 and is accompanied by CTCF PARylation (Figure 4F).

### CTCF PARylation is underpinned by the activity of the β-NAD^+^ salvage pathway

**Figure 5:**
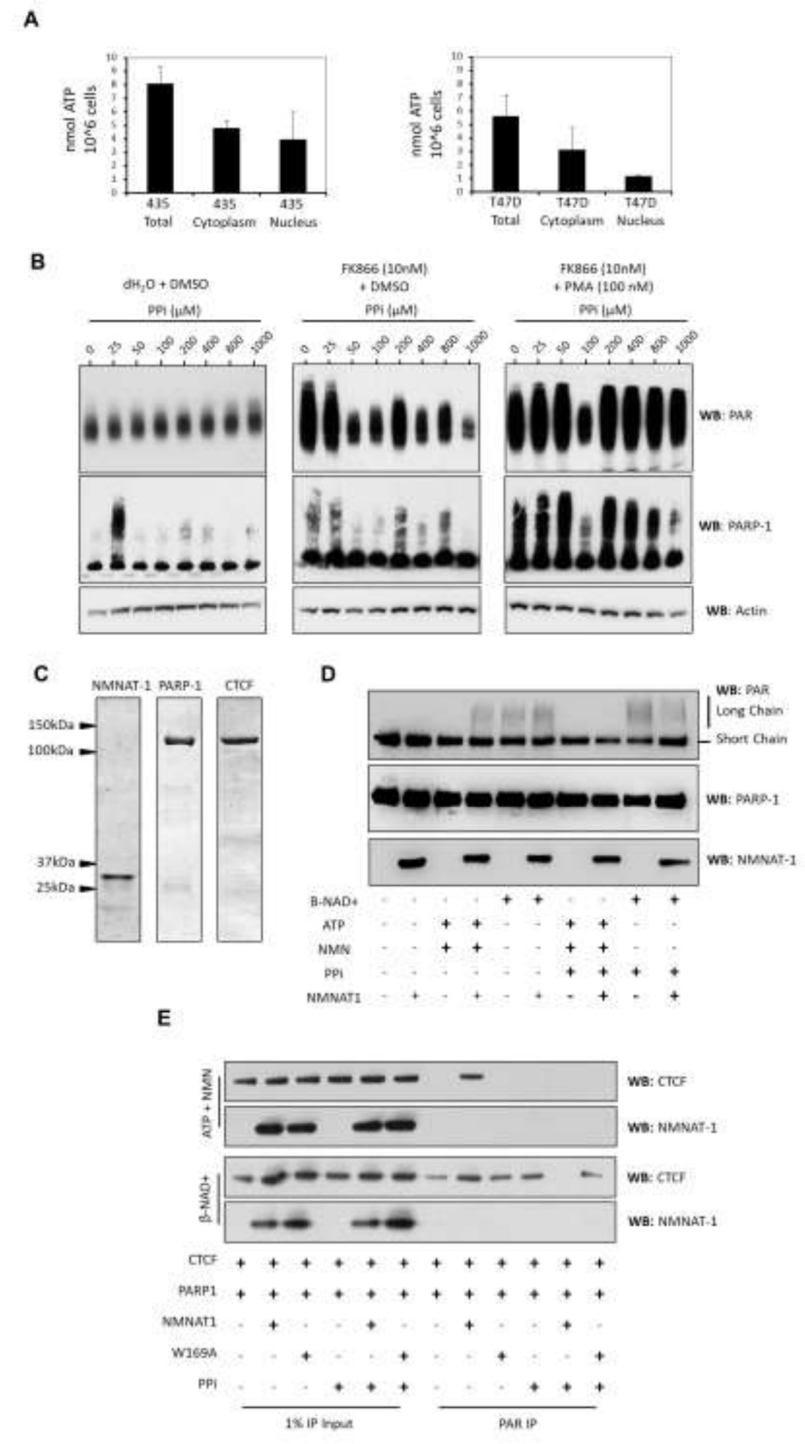
*In vitro* reconstitution of CTCF/PARP interaction and PARylation **A.** ATP levels were assessed by luciferase assay in 435 and T47D cell lysates. Total, cytoplasmic and nuclear concentrations of ATP were measured. 435 cells (upper panel) were observed to contain higher levels of ATP when compared to T47D cells. The levels of ATP were distributed evenly between cytoplasm and nuclear compartments. T47D cells (lower panel) displayed a slightly reduced level of total ATP compared to 435 cells. Furthermore, ATP concentrations were distributed unevenly between cytoplasm and nuclear compartments, where the nuclear levels of ATP were lower than cytoplasmic levels. **B.** β-NAD^+^ response assays conducted using nuclear lysates from T47D cells. Increasing pyrophosphate concentrations had little effect upon PARP activity without the NAMPT inhibitor FK866 (left panels). Addition of FK866 to the nuclear lysates enabled a dose-dependent decrease in PARP activity in response to pyrophosphate (middle panels). The FK866-induced sensitivity to pyrophosphate was ablated by pretreatment of cells with PMA (right panels). NAMPT activity potentially underpins the directionality of the NMNAT-1 reaction in T47D nuclear lysates. **C.** Recombinant protein preparations used in this study are visualized by coomassie staining. **D.** *In vitro* PARP response assay. Western blot for PAR showing short chain and long chain PARylation of PARP-1 (upper panel). Western blots for PARP-1 and NMNAT-1 (lower two panels). PARP-1 response with and without NMNAT-1 showing that NMN and ATP can be catalyzed into β-NAD^+^ by NMNAT-1 (lane 4) and that pyrophosphate inhibits this synthesis of β-NAD^+^ (lane 8). β-NAD^+^ addition leads to the auto-PARylation of PARP-1 with or without NMNAT-1 (lanes 5 and 6) but in the presence of NMNAT-1, pyrophosphate reduces the synthesis of long chain PAR polymers on PARP-1 (lane 10). **E.** CTCF PARylation assays. Input protein (first 6 lanes) and PAR IP (lanes 7-12) showing the PARylation of CTCF in reactions containing CTCF/PARP-1/NMNAT-1. Upper two panels: NMN and ATP can be metabolized to β-NAD^+^ by active NMNAT-1, leading to CTCF PARylation (lane 8) but not by a catalytically inactive mutant NMNAT-1 (lane 9). β-NAD^+^ synthesis is inhibited in the presence of pyrophosphate, preventing CTCF PARylation (lane 11). Lower two panels: β-NAD^+^ addition leads to the PARylation of CTCF in all instances other than when active NMNAT-1 and pyrophosphate catalyze the pyrophosphylitic cleavage of β-NAD^+^, thus significantly inhibiting CTCF PARylation (lane 11). This effect was not observed with the inactive mutant of NMNAT-1 (lane 12).

To further investigate how the β-NAD^+^ salvage pathway may affect CTCF PARylation we examined the catalytic and biochemical properties of PARP-1 and NMNAT-1 when complexed with CTCF. NMNAT-1 can catalyze both the forward and reverse reaction resulting in the synthesis or degradation of β-NAD^+^ (Figure 1C). It is generally accepted that the reverse reaction is limited *in vivo* by the availability and binding of pyrophosphate (PPi), where the abundance of ATP drives the synthesis of β-NAD^+^ [25, 26]. We assessed the intracellular levels of ATP in 435 and T47D cells using a luciferase-based ATP assay. We found that ATP levels were similar between cytoplasmic and nuclear fractions isolated from 435 cells but were reduced in the nucleus of T47D cells when compared to the bulk cytoplasm (Figure 5A). When combined with the downregulation of NAMPT and β-NAD^+^ levels (Figure 1), this observation suggests that the synthesis of β-NAD^+^ may be impeded and the metabolic equilibrium could shift toward the left of the NMNAT-1 catalyzed activity. We therefore investigated whether increasing PPi concentrations might impact the activation of PARP in T47D cell lysates. We found that increasing PPi concentrations alone had little effect on PARP activity (Figure 5B, left panels). However, further suppression of NAMPT with the inhibitor FK866 led to an increased sensitization to PPi and a dose-dependent suppression of PARP activity (Figure 5B, center panels) suggesting that the downregulation of NAMPT activity may modulate the balance of NMNAT1 kinetics and PARylation.

To explore CTCF PARylation in detail we reconstituted an *in vitro* system through which we could interrogate the mechanistic details of how CTCF is enzymatically modified. Recombinant PARP-1, CTCF and NMNAT-1 were expressed and purified from mammalian (NMNAT-1) or insect (CTCF, PARP-1) cells in an effort to recapitulate the native structure and post-translational modification of the recombinant proteins (Figure 5C). We first ensured that the purified enzymes were catalytically active by examining PARP-1 auto-modification in response to NMN, ATP and β-NAD^+^. Sheared DNA was not added to the reactions, so as not to invoke the DNA damage response inherent to PARP-1 activity. We anticipated that the nuances of any NMNAT-1 effects on protein-specific PARylation would be more readily observed on basal PARP-1 activity when activated by the association with CTCF and NMNAT-1 [27]. We found that NMNAT-1 was catalytically active and synthesized β-NAD^+^ from NMN (200μM) and ATP (200μM) by measuring the resulting auto-modification of PARP-1 (Figure 5D). NMNAT-1-dependent PARylation of PARP-1 was ablated by the inclusion of an excess of pyrophosphate (500μM) in the reaction buffer (Figure 5D). Direct addition of β-NAD^+^ to the reactions resulted in auto-modification of PARP-1 in an NMNAT-1-independent manner. This was unaffected by pyrophosphate in the absence of active NMNAT-1, thereby demonstrating that pyrophosphate levels used in these assays had no effect upon PARP activity. However when catalytically active NMNAT-1 and pyrophosphate were included in the reaction, the synthesis of long-chain PARP-1 auto-modification was reduced (Figure 5D).

Having substantiated that NMNAT-1 activity can induce or reduce PARP-1 auto-modification depending upon the metabolic context, we then examined whether CTCF PARylation could be modulated by the same process. To investigate the modification status of CTCF we first immunoprecipitated PAR, and then specifically assessed CTCF PARylation by western blot (Figure 5E). The addition of NMN and ATP to NMNAT-1/PARP-1/CTCF complexes resulted in the short chain PARylation of CTCF. This indicated that NMNAT-1-mediated β-NAD^+^ synthesis was sufficient to facilitate PARP-1 activity and modification of specific protein substrates (Figure 5E). This reaction was inhibited by inclusion of pyrophosphate thereby suggesting that NMNAT-1 not only affects PARP-1 auto-modification, but regulates modification of substrate proteins with short chain PAR.

To expand this hypothesis we then investigated if the pyrophosphylitic cleavage of β-NAD^+^ by NMNAT-1 can regulate CTCF PARylation. We directly added β-NAD^+^ to the *in vitro* protein complexes with and without pyrophosphate and again assessed the PARylation status of CTCF by PAR immunoprecipitation. Reactions incubated with β-NAD^+^ resulted in PARP-1-catalyzed CTCF PARylation, which was attenuated by the addition of pyrophosphate only when active NMNAT-1 was present. Using an inactive mutant of NMNAT-1 (W169A [24, 28]), we also observed that the catalytic activity of NMNAT-1 was required to inhibit CTCF PARylation (Figure 5E). In contrast to PARP-1, no smearing of CTCF protein bands was detected indicating that short chain PARylation of CTCF is the predominant form of modification in these DNA-independent reactions.

## DISCUSSION

CTCF binds to DNA elements throughout the genome by virtue of its 11 zinc finger domains and regulates many aspects of chromatin biology through association with various protein cofactors [11, 29, 30]. CTCF PARylation is known to facilitate its epigenetic boundary and insulator functions and regulate association of distinct protein interaction partners such as nucleolin [9, 10, 19]. It is therefore important to understand the mechanisms that modulate the PARylation status of CTCF to gain insight into how genomic boundary patterns are regulated.

Our study focused on comparing two human cancer cell lines (435 and T47D) to explore the regulation of CTCF PARylation because of stark differences in PARP activity and protein PARylation, which is associated with differential stability of CTCF-dependent chromatin boundaries adjacent to tumor suppressor genes [19]. It is important to note that these two cell lines do not exhibit the dual PARylated variants of CTCF (130kDa and 180kDa forms) that have been described in the literature (Figure 1B, Supplementary Figure S1). Both the T47D and 435 cell lines appear to contain only the 130kDa form of CTCF, which is perhaps unsurprising given that these are advanced breast cancer cell lines [20]. Nevertheless, PARylation of this form of CTCF has been reported [18].

On a molecular level, the stable association of PARP-1 and CTCF in T47D cells provides an interesting and relevant model in which to examine the regulation of active and inactive PARP complexes. Working under the hypothesis that the stable association of these proteins represents a non-productive reaction intermediate, we have endeavored to expand upon current knowledge about CTCF PARylation by examining the metabolic context and cellular signaling systems which underpin its regulation.

**Figure 6:**
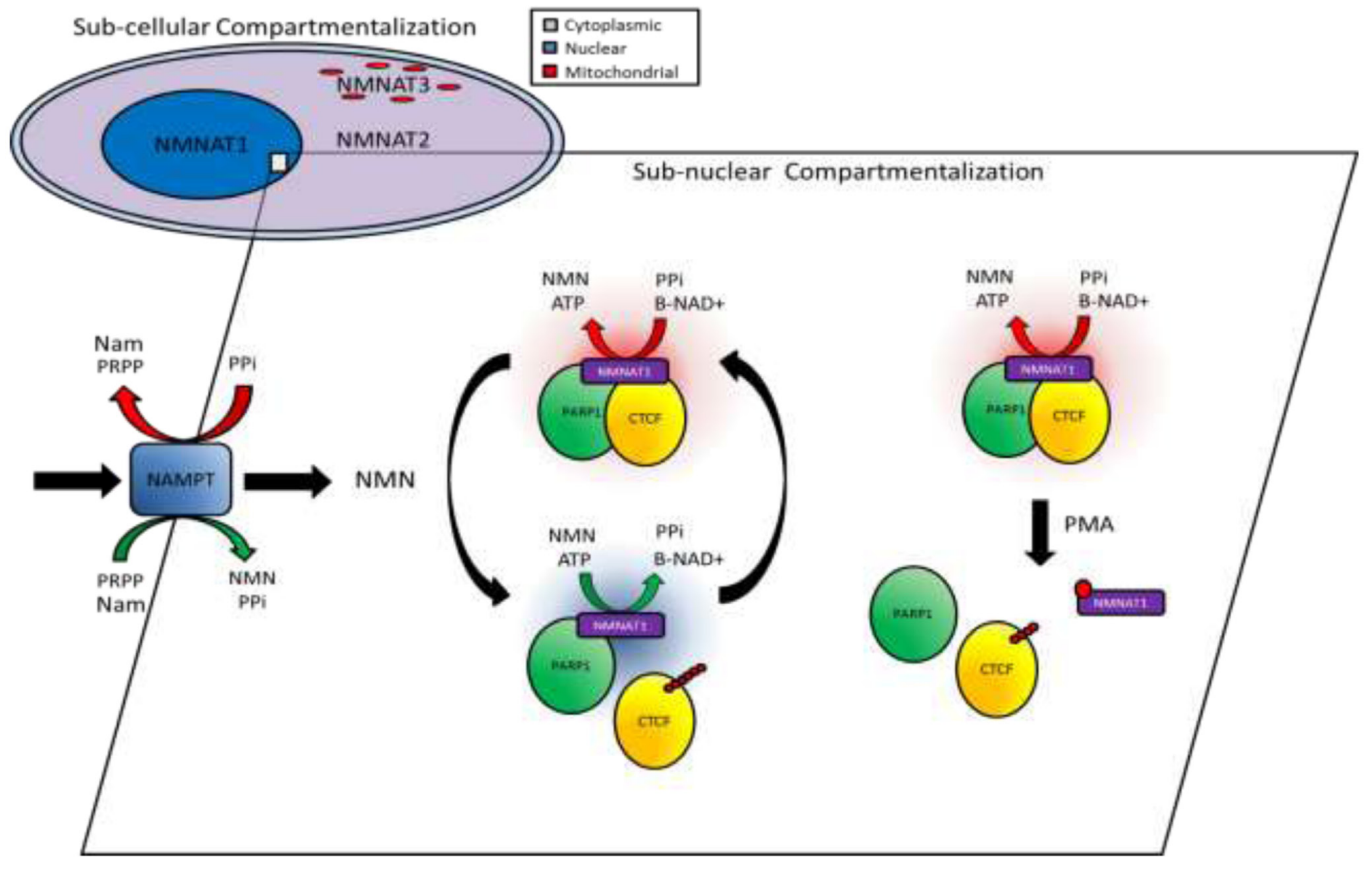
Schematic diagram of the proposed role for NMNAT-1 and the β-NAD^+^ salvage pathway in underpinning compartmentalized PARP dynamics and CTCF PARylation Subcellular localization of NMNAT enzymes underpins the first layer of compartmentalized supply of β-NAD^+^. Within the nucleus, alternative enzyme kinetics of NMNAT-1 regulate the second layer of protein complex-specific β-NAD^+^ availability whereby metabolite concentration and post-translational modification of NMNAT-1 can modulate enzymatic activity and lead to activation or inhibition of PARP complexes potentially resulting in dynamic regulation of CTCF PARylation.

The enzymatic activities of NAMPT and NMNAT-1 are critical regulators of the level and localization of nuclear β-NAD^+^ synthesis and are therefore pivotally placed to mediate β-NAD^+^-dependent PARP activity [21, 23, 31]. Interestingly, the low expression of NAMPT in T47D cells has previously been reported [32]. Down-regulation of this rate-limiting enzyme is sufficient to explain the observed reduction in nuclear availability of β-NAD^+^. However, it is widely accepted that subcellular compartmentalization and protein complex-directed supply of β-NAD^+^ depends upon the subcellular localization patterns of the NMNAT enzymes [21, 24, 28, 33] (Figure 6). We show how down-regulation of NAMPT transcription is combined with decreased nuclear NMNAT-1 protein stability, resulting in a reduced nuclear pool of β-NAD^+^. Within this context a direct relationship between CTCF and the β-NAD^+^ salvage pathway exists where a pool of NMNAT-1 (despite its down-regulation) remains associated with poised CTCF/PARP-1 complexes. These complexes remain enzymatically non-productive and the proximity of the β-NAD^+^ synthesis enzyme is insufficient to stimulate CTCF modification. Furthermore, this pool of bound NMNAT-1 is stable despite a general proteasome-mediated down-regulation of NMNAT-1.

As described in other systems, treatment with the PKC activator PMA resulted in a significant increase in PARP activity, PAR synthesis and the phosphorylation of NMNAT-1 in T47D cells [24]. This was accompanied by restoration of CTCF PARylation, dissociation of the CTCF/PARP-1/NMNAT-1 protein complex, and decreased NMNAT-1 protein stability over that observed in resting T47D cells. Akin to the mechanism that maintains low levels of NMNAT-1, the PMA-induced destabilization was also found to be proteasome-dependent. We hypothesize that T47D cells actively suppress protein PARylation by targeting specific pools of NMNAT-1 for degradation, where activated NMNAT-1 (in actively PARylating protein complexes) is subject to degradation resulting in the passive reduction of PARP activity through dissociation of localized β-NAD^+^ synthesis. This would explain why NMNAT-1 remains stably bound to inactive PARP-1/CTCF complexes despite reduced total levels of NMNAT-1 protein and PARP activity in T47D cells. As well as increasing PARP activity, PMA treatment has been shown previously to stimulate phosphorylation of NMNAT-1, resulting in its dissociation from PARP-1 complexes [24]. Our results show that this phosphorylation coincides with loss of NMNAT-1 from PARP-1/CTCF complexes and subsequent ubiquitination and degradation of the protein. These combined observations suggest that degradation of NMNAT-1 completes a mechanistic feedback loop that controls termination of localized pro-PARylation signaling. In T47D cells this feedback loop is overactive, resulting in a general reduction in PAR synthesis. However, the stable association of NMNAT-1 with inactive PARP-1/CTCF complexes suggests a direct suppression of PARP-1 activity rather than the proteasome-mediated NMNAT-1 feedback loop being solely responsible for modulation of PARP-1 activity.

NMNAT-1 has been shown to inhibit synthesis of branched PAR chains and general PARP-1 activity in biochemical reactions using high concentrations of recombinant protein. Moreover, the enzymatic activity of NMNAT-1 can facilitate both pyrophospholytic cleavage of β-NAD^+^ as well as synthesis of the metabolite [24, 34]. In an isolated system the equilibrium constant for NMNAT-1 activity is reported to be 0.3, thereby favoring cleavage of β-NAD^+^ [25, 26, 35]. A recent report strengthens the biological relevance of this observation by implicating ATP synthesis (a product of the pyrophospholytic cleavage of β-NAD^+^) by NMNAT-1 in ATP homeostasis in WldS axonal protection [36]. When considering NMNAT-1 activity in T47D cells, down-regulation of NAMPT suggests a metabolic context where NMN synthesis limits the NMNAT-1 catalyzed production of β-NAD^+^. Indeed, further suppression using an inhibitor of NAMPT activity increases the suppression of PARP activity observed in response to elevating PPi concentrations. Our biochemical reactions using purified proteins suggests that under the correct metabolic conditions NMNAT-1 catalyzed pyrophospholytic degradation of β-NAD^+^ is capable of disrupting protein PARylation by PARP-1. However, further study is required to determine whether this activity is a mechanism that regulates localized patterns of specific protein PARylation and protein function within the more complex metabolic environment of the cell nucleus.

PARP-1 activity is stimulated in response to cellular stress and is a key factor in driving cell fate decisions toward survival or apoptosis. Alongside this, stress also upregulates NMNAT-1 expression in normal mammalian and Drosophila cells [37, 38]. This is presumably to support the enhanced activity of PARP-1 and avert apoptosis induced by the depletion of β-NAD^+^ during prolonged hyperactivation of PARP enzymes. In many types of cancer cells PARP-1 activity and expression are upregulated [39] and essential to support cell survival and promote the aberrant proliferative phenotype. However, we and others have observed that NMNAT-1 becomes down-regulated in many cancer cell lines and tumors [38] (data not shown). The contrasting changes in the expression and activity of PARP-1 and NMNAT-1 in cancer cells suggest that differential modulation of the β-NAD^+^ salvage pathway is part of a complex cellular program which results in the disruption of localized PARP-1 activity while maintaining a global increase in the proliferative phenotype and cell survival. The loss of CTCF PARylation may result in aberrant regulation of chromatin boundaries as an extension of this program and the resulting changes in epigenetic programming promote aberrant gene silencing. This would add further selective pressure to favor increasingly aggressive phenotypes and a possible pathway for cancers to evolve as tumor suppressor loci become silenced in response to the loss of CTCF PARylation [19].

## MATERIALS AND METHODS

### Molecular biology

Plasmids used in these studies were prepared in DH5α E. Coli and purified using Maxiprep kits sourced from Life Technologies. pCMV5-NMNAT-1, pCMV5-NMNAT-1W169A and pCMV5-PARP-1 were kind gifts from Dr. Lee Kraus.

### RT-qPCR and gene expression analysis

RNA was extracted from cells using Trizol (Life Technologies) and first-strand cDNA was synthesized using Superscript III reverse transcriptase (Life Technologies) as per the manufacturer’s instructions. Thermocycling and analysis was conducted with an ABI Prism 7300 qPCR analyzer using ABI Sybr Green Mastermix (Life Technologies).

### Protein biochemistry

Whole cell lysates were extracted from cells using a high salt modified RIPA buffer (50mM Tris pH7.5, 400mM NaCl 0.1% SDS, 0.5% Sodium deoxycholate, 0.5% NP-40) and lysed on ice for 30 minutes before centrifugation at 14,000xg for 30 minutes. Nuclear lysate preparations were extracted from cells by the two-step method using hypotonic lysis of the plasma membrane followed by high salt extraction of nuclear proteins. Briefly, cells were harvested by trypsinization and washed with PBS. The resulting cell pellet was incubated in a hypertonic lysis buffer (10mM HEPES pH 7.5, 10mM KCl) for 10 minutes before the addition of NP-40 to 0.1%. The cell suspension was agitated and centrifuged at 800xg for 1 minute to pellet nuclei. Nuclei were suspended in two pellet volumes of nuclear extraction buffer (10mM HEPES pH 7.5, 1mM MgCl, 400mM NaCl, 0.5mM EDTA). All lysis buffers were supplemented with protease and phosphatase inhibitor cocktails (Roche) and DTT (1mM). Immunoprecipitations were performed with lysates diluted to 150mM NaCl using dilution buffer (10mM HEPES pH 7.5, 1mM MgCl, 0.5mM EDTA). 3μg of rabbit CTCF antibody (Active Motif) was incubated with lysates and bound protein complexes were precipitated using protein G Dynabeads (Life Technologies), washed three times with wash buffer (20mM HEPES pH 8, 1.5mM MgCl, 150mM NaCl, 0.5mM EDTA, 0.3% Triton X100) and visualized using western blotting. For PAR IP and phospho-serine IP, 30µl of clone 10H anti-poly(ADP)ribose agarose (Tulip Biolabs) or anti-phospho-serine agarose (mouse ab, Sigma-Adrich) beads were used to precipitate PARylated proteins. Western blot analysis was carried out post-protein separation by SDS-PAGE over a 4-12% or 10% Bis-Tris precast protein gel (Life Technologies) using MOPS running buffer (Life Technologies) and wet transferred to nitrocellulose membranes. Membranes were either imaged using X-ray film or a Chemidoc touch (Biorad, USA) imaging system. Mouse monoclonal G8 CTCF antibody (Santa Cruz Biotechnology), mouse monoclonal F1 PARP-1 antibody (Santa Cruz Biotechnology), mouse monoclonal NMNAT-1 antibody (Santa Cruz Biotechnology), rabbit polyclonal NAMPT (PBEF) (Abcam), and mouse monoclonal 10H poly(ADP)ribose (Tulip Biolabs) were used in western blotting procedures. HRP secondary antisera were sourced from Santa Cruz Biotechnology.

### Cell culture

MDA-MB-435, T47D cells and HEK293T cells were sourced from ATCC and grown in RMPI1640 (435, T47D) or DMEM (HEK293T) culture media supplemented with 10% fetal bovine serum (Omega, USA). HEK293T cells stably expressing wild type NMNAT-1 were created following transfection and spontaneous incorporation of the sequence into the cellular genome. Selection was performed using puromycin at 100ng/ml. Transfections were carried out using a calcium phosphate based procedure or using xtreamgene 9 reagent from Roche. PMA and MG132 used in cell culture were sourced from Sigma. Cells were harvested after 5 hours of MG132 and/or PMA treatment unless annotated otherwise.

### PARP response assay

100ug of nuclear protein extract was diluted to a concentration of 1µg/µl and incubated with 1mM β-NAD^+^ for 30 minutes at 4°C (10mM HEPES pH 7.5, 1mM MgCl, 150mM NaCl, 0.5mM EDTA). Reactions were terminated by addition of SDS loading buffer and heating to 100°C for 3 minutes. Sample PARylation was assessed by western blot using clone 10H PAR antibody (Tulip Biolabs). 3-Aminobenzamide (3’-ABA) used in this study was obtained from Sigma (USA).

### Fluorescent metabolite incorporation

Fluorescently labeled β-NAD^+^ β-Nicotinamide- N^6^- (2- (6-[fluoresceinyl]aminohexanoyl)aminoethyl) adenine dinucleotide (F*Β-NAD^+^) was obtained from Biolog (Germany). Whole cell nuclei were harvested as described above and incubated with 100μM F* β-NAD^+^ or F*ATP in transport buffer (20mM HEPES pH 7.3, 2mM MgAc, 5mM NaAc, 150mM KAc, 0.5mM EGTA) with or without 3’-ABA PARP inhibitor (Sigma) for 30 minutes at 4^°^C. Nuclei were stained with DAPI and the resulting nuclear suspensions were mounted using Immu-mount (Fisher Scientific). Nuclei were imaged using a Zeiss LSM 780 confocal microscope equipped with 20x and 63x objective lenses and the resulting images exported as *.tiff files and processed using ImageJ. Pseudocolor Images were created using the ’16 Color’ look-up-tables within ImageJ. Nuclear incorporation of fluorescent metabolites was quantified using the intensity measurement function of ImageJ and displayed as [mean intensity]/[area] relative fluorescence units. Students t-test was used to assess statistical significance.

Incorporation of fluorescent β-NAD^+^ Images for z-stack was recorded as for single immunofluorescence images in 1μm step-wise increments between frame captures. Frames were processed using ImageJ, and *.avi files generated using the ‘3D Project’ algorithm of the application. The frames were interpolated over 5 pixel spacing between each image to generate the 3D reconstructions.

### *In vitro* PARylation reactions

NMNAT-1 protein was purified from stable HEK293T-NMNAT-1 cells using a commercial FLAG purification kit (Sigma-Aldrich). W169A NMNAT-1 mutant protein was purified from transfected HEK293T cells using the anti-FLAG procedure as stable cell lines could not be generated with the mutant. CTCF was purified from SF12 insect cells by a twstage purification system using a poly-histidine and streptavidin-based protocol. For HIS purification Nickel-NTA resin was sourced from Qiagen and streptavidin affinity resin was purchased from Thermo Fisher. Briefly, insect cells were harvested in lysis buffer (50mM PO_4_, 500mM NaCl, 20mM imidazole, 1% IGEPAL CA-630, pH 7.5, 100mM PMSF) and treated with DNaseI (Roche) for 30 min on ice. Lysates were allowed to mix with 2 ml (per 50 ml lysate) of Nickel-NA resin for 30 min before washing three times (50mM PO_4_, 500mM NaCl, 20 mM imidazole, pH 7.5). Resin-bound proteins were eluted (50mM PO_4_, 500mM NaCl, 200mM imidazole, pH 7.5.) and incubated with Streptavidin resin for 30 min. Streptavidin-bound protein was then washed three times (50mM PO_4_, 500mM NaCl, pH 7.5) and eluted (50mM PO_4_, 200mM NaCl, 0.25mM desthiobiotin, pH 7.5). All buffers used during the purification procedure were supplemented with protease cocktail inhibitors (Roche) and 5mM β-mercaptoethanol. Eluted proteins were snap frozen and stored at -80°C before use. Recombinant PARP-1 was obtained from Tulip Biolabs. All *in vitro* reactions were carried out using 200 ng of CTCF, PARP-1 and NMNAT-1. Required proteins were combined in a total volume of 25 µl in PARP reaction buffer (20mM HEPES pH 8, 2mM MgCl_2_, 150mM NaCl, 0.5mM EDTA) and incubated at 37^°^C for 15 min to allow proteins to associate. Total reaction volumes were raised to 100 µl with reaction buffer supplemented with required small molecule reaction components: β-NAD^+^ (200μM where indicated, Sigma-Aldrich), nicotinamide mononucleotide (200µM, Sigma-Aldrich), ATP (200µM, Sigma-Aldrich) or tetrasodium pyrophosphate (500µM, Sigma-Aldrich). Reactions were then incubated for 10 min at 37^°^C. Reaction samples for immunoprecipitation experiments were raised to 500 µl with IP buffer (20mM HEPES pH 8, 2mM MgCl_2_, 150mM NaCl, 0.5mM EDTA, 0.5% NP-40) supplemented with PARP inhibitor 3’ABA (1 µM, Sigma-Aldrich) to terminate any further PARylation during the immunoprecipitation procedure.

### Metabolite extraction and ATP assay

Cells were biochemically fractionated as described above. ATP was extracted using a -20°C methanol/chloroform extraction method from cell fractions [40]. This was done to inactivate and separate ATP from enzymes which would otherwise deplete the metabolite in lysate assays. The aqueous phase was collected and dried under vacuum before resuspension in dH_2_O. Luciferase-based ATP assays were conducted as per the manufacturer’s instructions (Sigma-Aldrich).

### Alcohol dehydrogenase-coupled MTT assay for β-NAD^+^

The measurement of (NAD^+^/NADH) was performed as previously described [41]. All materials including recombinant alcohol dehydrogenase were sourced from Sigma-Aldrich.

## SUPPLEMENTARY MATERIALS FIGURES AND VIDEOS






