# Supplementary material for: The β-NAD+ salvage pathway and PKC-mediated signaling influence localized PARP-1 activity and CTCF Poly(ADP)ribosylation
Source: Oncotarget. 2017 Aug 3;8(39):64698–713. doi: 10.18632/oncotarget.19841 (PMC5630287; doi:10.18632/oncotarget.19841)
Supplement: Supplementary file 1 [file oncotarget-08-64698-s001.pdf]

The β-NAD+ salvage pathway and PKC-mediated signaling influence localized PARP-1 activity and CTCF Poly(ADP) ribosylation

SUPPLEMENTARY MATERIALS

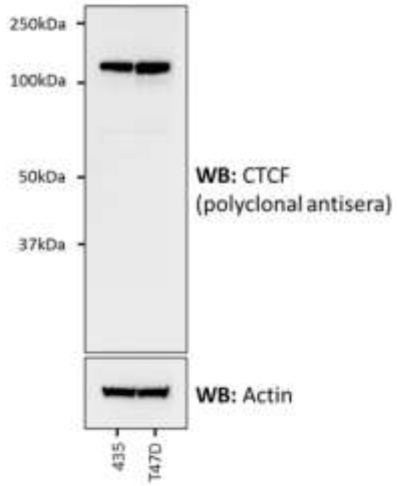

Supplementary Figure 1: CTCF polyclonal antisera recognized only the 130kDa form of CTCF in both the 435 and T47D cell lines. Actin was used as a loading control.

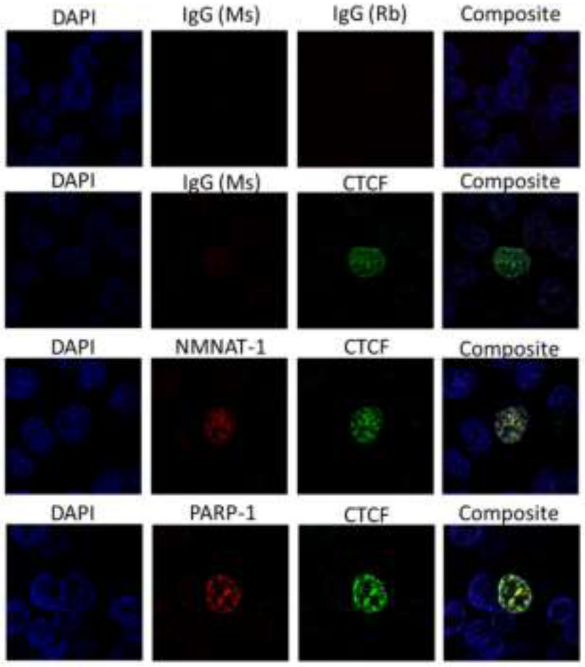

Supplementary Figure 2: Transfection of CTCF, PARP-1 and NMNAT-1 into T47D cells showed that all three proteins localized to similar regions within the nucleus. Mouse and Rabbit nonspecific IgG antisera were used as negative controls for background staining.

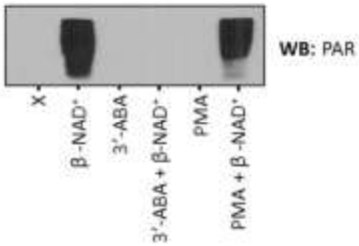

Supplementary Figure 3: PMA treatment (100nM) has no effect in T47D nuclear lysates.

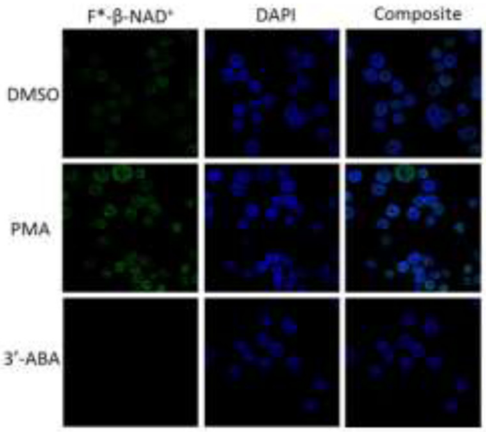

Supplementary Figure 4: PMA treatment increases PARP activity in T47D nuclei consistent with lysate-based studies. 3'-ABA was used to inhibit PARP activity and highlight PARP dependency of incorporation.

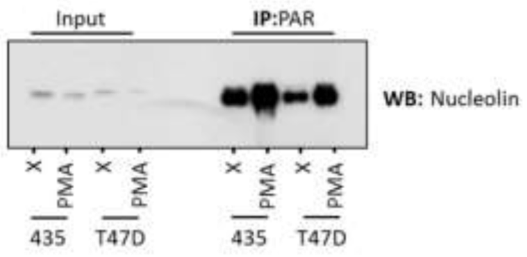

Supplementary Figure 5: PMA treatment stimulates nucleolin PARylation as assessed by immunoprecipitation of PAR and western blot for nucleolin in 435 and T47D cells.

For Supplementary Media files see in Supplementary Files.
